# Everybody's Page

**Published:** 1890-09-20

**Authors:** 


### EVERYBODY'S PAGE.

A serious case—A doctor without one.

---

The National Women's Health Association of America was organised in July, at Philadelphia, Dr. Caroline Dodson being president.

---

A good many people spoil the effect of a good night's rest by the ridiculously heavy bed-clothes they use. Old-fashioned cotton quilts or modern Massala ones are very heavy and no use, as a thin covering to protect blankets from the dirt is all that is really necessary. Bed-clothes should be like body clothing, light and warm. Many a bad sleeper would do well to see whether his coverings are not at the bottom of his restless nights.

---

Some people's consciences are of a peculiar build, particularly as regards "stamped envelopes for reply." It is a little hard on a trusting nature who, writing to ask politely for some information, encloses a stamp and gets neither a reply nor his stamp back again. There was once an old gentleman who went in for philanthropy, and he was overheard to remark to his clerk: "John, all those appeals for help enclosed a stamped envelope, I believe?" "They did, sir." "Then answer them all on halfpenny post-cards."

---

"What with electricity, massage, air-pumps, and toilet cupping, a really old woman bids fair to be a novelty," says the *Nightingale*. And if all these treatments have to be used to prevent the wear and tear of age, we think, on the whole, we prefer to submit to the ravages of time. After all, how far sweeter the women look who are content to believe that youth won't last for ever, compared to those who strive to be young for ever. Nature knows best. Art can do a good deal, but she can't make an old woman look young, but she can, and does, make a good many look ridiculous.

---

The following advertisements, all from one issue of the *Chicago Tribune*, make us doubt the proverbial 'cuteness of our cousins across the pond:—

CLAIRVOYANTS.

At once know the future—Consult Mrs. Franks, 541, Wabash-av., on love, marriage, divorce, lawsuit. Ladies, 50c. and $1. Always at home; 3 questions answered by mail, 50c. in stamps.

"Dr. Paul," world-renowned astrologer and clairvoyant, 427, West Madison-st., third floor, late of New York; seventh son; 30 years' practice; all affairs; true life horoscope. 50c. Hours, 9 to 5.

Louise Bennett, clairvoyant—Massage and magnetic treatment. 46 S., Clark-st., Room 10.

Mrs. L. Pet Anderson, trance medium; nativities given. 30, Ogden-av., near Randolph.

Mme Joy, the famous clairvoyant fortune-teller; 50c. and $1. 445, West Madison-st.

Madame Villers, 50, West Madison-st.—True card reading. Always home. Satisfaction.

The great fortune teller—The young Madame Carrie L. Lamont, the great astrologist, has travelled through the principal parts of Europe, will remain 30 days; tells past, present, and future in person or by letter; will bring back the parted husband or lover, no matter if they be 10,000 miles away; will tell you whether your lover be false or true; will guarantee to settle family quarrels, or money refunded; can also cause speedy marriages; has charms for good luck; breaks evil influence; gives lucky numbers in lottery; can give best of reference in regard to above statement. I was presented with elegant gold medal in Colorado. Can read the initials of a caller's name by their hand. Ladies in trouble, call. All business confidential. Office hours, 10 a.m. to 9 p.m. Madame C. L. Lamont, parlour upstairs, Room 1, 515, Wabash-av., cor. Harmon-court, Chicago, Ill.

Professor Koch is about to begin experimentalising on living persons his treatment for the cure of tuberculosis.

---

Homœopathy must be popular in Boston—50,000 dollars are forthcoming wherewith to build a new homœopathic dispensary.

---

The National Health Society is going to repeat the lectures to ladies on personal and domestic hygiene, under the patronage of H.R.H. the Duchess of Albany. The address is now 53, Berners Street, Oxford Street.

---

"Pluck" is one of the things that few English lads are wanting in. Three good examples were the boys, aged respectively thirteen, fourteen, and fifteen, who obtained medals on September 10th from the Royal Humane Society for bravery in saving life from drowning.

---

The *Hospital Gazette* is said to have solved the secret of British thirst, viz., that the annual consumption of salt per head in England exceeds that of other European countries! Query? We should say the homely pipe of tobacco accounted for a great part of the British thirst.

---

That statistics are often a blind guide is fairly clear, yet the steady diminution of the death-rate in this country during the past few years must point to some efficient cause. Sanitary reformers may well claim some share in this result, and there can be little doubt that compulsory notification of infectious diseases, which puts preventive measures within the power of local authorities, will considerably strengthen their hands.

---

"Ill-health," says Sir John Lubbock, "is no excuse for moroseness. If we have one disease, we may, at least congratulate ourselves that we are escaping all the rest. Sydney Smith, ever ready to look on the bright side of things, once, when borne down by suffering, wrote to a friend that he had gout, asthma, and seven other maladies, but was 'otherwise very well,' and many of the greatest invalids have borne their sufferings with cheerfulness and good spirits."

---

If vegetarianism were universal, three men could live where one lives now, likewise six men could live where one horse now exists. At least so it is asserted. We feel very dubious as regards the veracity of this statement, and for our part prefer the happy medium. A war waged against excessive meat eating, and a sermon preached on the purifying part which vegetables might be allowed to play in our internal economy, would, to our minds, be a useful undertaking. If Englishmen would learn part of the lesson of the vegetarian, if he would modify his supply of "roast beef," and increase doubly or trebly the supply of fresh vegetables and fruit at his table, we should hear fewer groans about indigestion, and to many insomnia would only remain as an unpleasant memory.

